# NFKB1 truncation–associated recurrent, sterile inflammation effectively treated with IL-1β blockade

**DOI:** 10.70962/jhi.20260157

**Published:** 2026-10-23

**Authors:** Kevin S. Ackerman, Isabel F. Armendi, Jay J. Mehta, Neil Romberg

**Affiliations:** 1 Division of Immunology and Allergy, Children’s Hospital of Philadelphia, Philadelphia, PA, USA; 2 Division of Rheumatology, Children’s Hospital of Philadelphia, Philadelphia, PA, USA; 3 Department of Pediatrics, Perelman School of Medicine, University of Pennsylvania, Philadelphia, PA, USA

## Abstract

Recurrent, sterile inflammatory responses to surgery and trauma in a patient with a heterozygous, truncating *NFKB1* variant are described. This case illustrates the autoinflammatory phenotype of truncating *NFKB1* variants and highlights the prospect of a targeted, personalized immunomodulatory therapy.

## Introduction

The nuclear factor κ light-chain enhancer of activated B cells (NFKB1) pathway is a central regulator of inflammatory gene expression. NFKB1 haploinsufficiency is an autosomal dominant disorder that can present with common variable immunodeficiency (CVID) and autoimmunity. Several patients with CVID and truncating *NFKB1* variants have also been reported to experience sterile inflammatory reactions, including pyoderma gangrenosum (PG), necrotizing fasciitis, cellulitis, postpartum poor wound healing, and sterile sepsis (1, 2, 3, 4). A recent mechanistic report by Nurmi et al. described how NFKB1 truncation impairs myeloid cell autophagy, aberrantly activates the NLRP3 inflammasome, and generates excessive IL-1β (1).

Here, we present the case of an 18-year-old male patient with CVID and multiple episodes of sterile inflammation who was ultimately and successfully treated with IL-1β blockade.

## History of autoinflammatory disease

**Figure 1.**
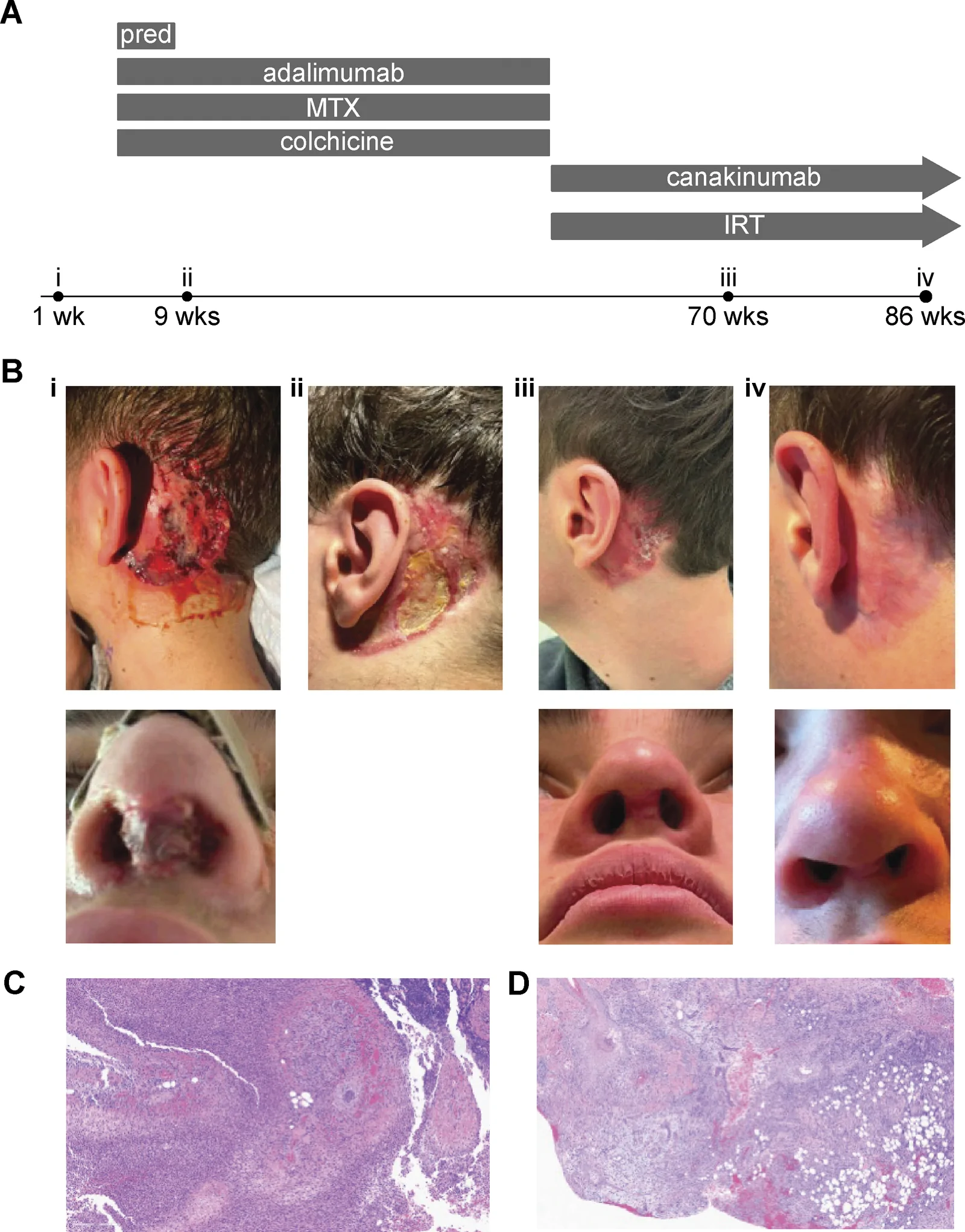
**Patient sterile inflammation and its treatment. (A)** A timeline of immunomodulatory therapies after surgical reconstruction is depicted. Time points i–iv indicate when clinical images were captured. **(B)** Images of the patient’s left postauricular area and anterior nose columellar graft are displayed at the following time points: (i) postoperative day 7 (nose) and 13 (ear); (ii) 9 wk after operation and 7 wk after initiation of Behçet’s-directed therapies; (iii) 70 wk after operation and 16 wk after initiation of canakinumab and discontinuation of other therapies; (iv) 86 wk after operation, 32 wk after initiation of canakinumab. **(C)** H&E stain of inferior left ear deep dermis (20×) displays deep dermal and subcutaneous neutrophilic inflammation. **(D)** H&E stain of the middle left ear epidermis to subcutis (10×) displays an ulcer with dermal and subcutaneous neutrophilic inflammation. pred, methylprednisolone/prednisone; MTX, methotrexate; IRT, immunoglobulin replacement therapy; wks, weeks.

As an infant in the neonatal intensive care unit, the patient developed an erosion of his nasal columella, which was attributed to prolonged contact with nasal respiratory support interfaces. At 15 years of age, to correct the longstanding nasal deformity, he underwent nasal reconstruction with a right ear graft that failed by 3 mo post-operatively with nasal tip collapse resulting in moderate nasal obstruction. A concurrent inguinal hernia repair was complicated by fevers and a purulent yet sterile abscess at the surgical site. He was treated with antibiotics for presumed infection, but no causative microorganisms were identified. 8 mo later, at 16 years of age, reconstruction of the full-thickness columellar defect with a left ear composite cartilage and cutaneous graft was complicated by a large, nonhealing skin ulcer at the graft site that was unresponsive to antibiotics and a surgical washout. Laboratory evaluation revealed leukocytosis, peak 28.9 K/μl (reference 3.8–9.8 K/μl) with elevated absolute neutrophil count, 21.1 K/μl (1.5–7.0 K/μl), elevated C-reactive protein, 12.0 mg/dl (0.0–0.9 mg/dl), and normal erythrocyte sedimentation rate. Pathologic evaluation of the skin wound debridement and biopsy revealed an ulcer with deep dermal and subcutaneous neutrophilic inflammation with negative microbial stains (Grocott-Gomori methenamine silver [GMS], Periodic acid-Schiff [PAS], acid-fast bacilli [AFB], Fite, gram, and mucicarmine), findings that are compatible with the clinical impression of PG (Fig. 1, C and D). In conjunction with a history of recurrent oral ulcers and a new purulent scrotal ulcer, his presentation was initially felt to be consistent with incomplete Behçet disease. He was treated with pulse-dose methylprednisolone followed by prednisone for 1 mo with a taper in addition to adalimumab, methotrexate, and colchicine (Fig. 1 A). Leukocytosis, neutrophilia, and elevated C-reactive protein resolved by the time of follow-up 4 wk post-operatively. However, he had incomplete skin healing over the ensuing months (Fig. 1 B). He also continued to have intermittent oral ulcers, fatigue, and an increased frequency of upper respiratory infections.

## History of infectious disease and hypogammaglobulinemia

The patient had recurrent acute otitis media requiring tympanostomy placement bilaterally and was admitted to the hospital at age 3 years with pneumonia and at 3.5 years with maxillary/ethmoid sinusitis. Immunologic evaluation during the latter admission was performed, which revealed low IgG, 390 mg/dl (reference 477–1,334 mg/dl), low IgA, 28 mg/dl (40–251 mg/dl), and low IgM, 40 mg/dl (51–194 mg/dl), with initially low tetanus and pneumococcal antibody levels, and prophylactic amoxicillin was initiated. 2 mo later, he had a normal vaccine response to DTaP and the pneumococcal polysaccharide vaccine 23 (PPSV23); IgG normalized to 530 mg/dl later, but IgA, 32 mg/dl, and IgM, 37 mg/dl, remained low. Infection frequency improved, but he was then lost to follow-up. IgA was noted to remain low at 28 mg/dl (70–456 mg/dl) and 25 mg/dl (52–221 mg/dl) at age 11 and 13 years, respectively, in the setting of celiac testing, but no other immunophenotyping was obtained in these intervening years. Immunologic evaluation starting at age 16 years 11 mo, after the multiple postsurgical complications, was notable for hypogammaglobulinemia with low IgG, 503 mg/dl (reference 671–1,456 mg/dl), low IgA, 25 mg/dl (90–386 mg/dl), normal IgM, 38 mg/dl (35–168 mg/dl), insufficient response to conjugated pneumococcal immunization, and low switched memory B cells (CD19^+^/CD27^+^/IGM^−^), 1.0% (3.9–30.7%), consistent with CVID. A review of lifetime infectious history was notable for frequent otitis media as a child requiring tympanostomy tube placement, around two sinus infections annually since early childhood, six pneumonias, three of which required hospitalization, and six to eight respiratory tract infections per year, which had increased to about every 3 wk after starting Behçet-directed immunosuppressive therapies.

## Genetic evaluation and personalized treatment

Due to his atypical clinical picture, a primary immunodeficiency–focused genetic panel was obtained and revealed a heterozygous pathogenic truncating *NFKB1* variant (NM_003998.4, c.909dup, p.Thr304Hisfs*5). Given published evidence of truncated NFKB1 aberrantly activating the NLRP3 inflammasome, and stagnation in skin healing, the IL-1β inhibitor canakinumab at 150 mg monthly administered subcutaneously was started, and adalimumab, methotrexate, and colchicine were discontinued. At the time of treatment modification, 54 wk post-operatively, the patient’s skin wounds appeared grossly similar to 9 wk post-operatively (Fig. 1 B ii). After initiation of canakinumab, the patient’s skin wounds significantly improved, and the frequency of oral ulcers decreased over the subsequent months (Fig. 1 B). Intravenous immunoglobulin replacement at 500 mg/kg was concurrently initiated due to newly identified CVID with resolution of fatigue, upper respiratory, and sinopulmonary infections.

## Discussion

We present one of the first published cases of NFBK1 truncation–mediated autoinflammation treated with targeted IL-1β inhibition. This case highlights how broad genetic evaluations of recurrent, sterile inflammation can generate valuable and actionable clinical information. One series of five adult patients with steroid-refractory PG without reports of genetic sequencing were treated with canakinumab; four patients had a decrease in target-lesion size and improvement in quality of life scores, and three achieved complete remission, highlighting the potential of IL-1β inhibition in PG pathology (5). Rather than relying upon empiric anti-inflammatory therapy regimens, a molecular diagnosis can provide individualized precision medicine opportunities. Although we observed remarkable and rapid healing of long-standing sterile inflammatory wounds in our patient, longitudinal monitoring will be required to determine if the therapeutic response is durable and potentially preventive of future episodes at new sites.

## Ethics statement

### Ethics and informed consent

Oral informed consent was obtained from the patient and guardian for publication of this case report, including publication of clinical images.

## Data Availability

The genetic *NFKB1* variant described here has been submitted to the ClinVar database and is assigned accession number SCV008095474.
